# Risk Factors and Prevalence of Suicide Attempt in Patients with Type 2 Diabetes in the Mexican Population

**DOI:** 10.3390/ijerph15061198

**Published:** 2018-06-07

**Authors:** Tania Guadalupe Gómez-Peralta, Thelma Beatriz González-Castro, Ana Fresan, Carlos Alfonso Tovilla-Zárate, Isela Esther Juárez-Rojop, Mario Villar-Soto, Yazmín Hernández-Díaz, María Lilia López-Narváez, Jorge L. Ble-Castillo, Nonanzit Pérez-Hernández, José Manuel Rodríguez-Pérez

**Affiliations:** 1 División Académica Multidisciplinaria de Comalcalco, Universidad Juárez Autónoma de Tabasco, Comalcalco 86025, Tabasco, Mexico; tanyperalta93@gmail.com; 2 División Académica Multidisciplinaria de Jalpa de Méndez, Universidad Juárez Autónoma de Tabasco, Jalpa de Méndez 86200, Tabasco, Mexico; thelma.glez.castro@gmail.com (T.B.G.-C.); yazmin.hdez.diaz@gmail.com (Y.H.-D.); 3 Subdirección de Investigaciones Clínicas, Instituto Nacional de Psiquiatría Ramón de la Fuente Muñíz, Ciudad de Mexico 14370, Mexico; fresan@imp.edu.mx; 4 División Académica de Ciencias de la Salud, Universidad Juárez Autónoma de Tabasco, Villahermosa 86140, Tabasco, Mexico; jblecastillo@hotmail.com; 5 Hospital de Alta Especialidad “Gustavo A. Rovirosa Pérez”, Secretaría de Salud, Villahermosa 86140, Tabasco, Mexico; mariovillarsoto@hotmail.com; 6 Hospital General de Yajalón “Dr. Manuel Velasco Suarez”, Secretaría de Salud, Yajalón 29930, Chiapas, Mexico; dralilialonar@yahoo.com.mx; 7 Departamento de Biología Molecular, Instituto Nacional de Cardiología Ignacio Chávez, Ciudad de Mexico 14080, Mexico; unicanona@yahoo.com.mx (N.P.-H.); josemanuel_rodriguezperez@yahoo.com.mx (J.M.R.-P.)

**Keywords:** suicide, depression, diabetes, Mexican population

## Abstract

Background: It has been proposed that the risk of death by suicide is higher in patients with diabetes than in the general population. Therefore, it is necessary to investigate the risk factors of suicidal behavior in patients with type 2 diabetes. The aim of the present study was to analyze the prevalence of suicide attempt and determine the risk factors of suicide attempt, in patients with type 2 diabetes in a Mexican population. Methods: Clinic characteristics, anthropometric measurements, biochemical levels, depression, and suicidal behavior were evaluated in 185 Mexican patients with type 2 diabetes. A multivariate logistic regression analysis was performed to find predictive factors of suicide attempt. Results: 11.4% of patients reported previous suicide attempts n = 21). Younger patients (OR: 3.63, 95% CI: 1.29–10.19), having depression (OR: 3.33, 95% CI: 1.13–9.76) and normal BMI (OR: 3.14, 95% CI: 1.11–8.83), were predictive factors of suicide attempt. No other variables in the study showed statistical significance. Conclusions: Our results showed a high prevalence of suicidal behavior in patients with type 2 diabetes. We found that younger age, depression and normal BMI could be risk factors of suicide attempt in these patients. Therefore, psychiatric interventions to prevent depression and suicidal behavior in this population are necessary. New studies using larger samples are necessary to replicate and confirm these results.

## 1. Introduction

Diabetes mellitus (DM) is a chronic disease that has become a serious public health problem worldwide [1,2,3]. Several studies support that DM has profound effects on physical and emotional health. Furthermore, DM has been associated with many adverse health effects including reduced life expectancy, increased risk of various complications, decreased quality of life, and even death [1,3,4,5].

On the other hand, it has been reported that patients with chronic medical conditions such as type 2 diabetes mellitus (T2DM), are more likely to manifest psychiatric traits when compared with healthy individuals [6,7,8,9]. Up to today, the cause and effect relationship between these entities is not clear yet; we do not know whether diabetes increases the risk of psychiatric traits as depression, or whether psychiatric traits increase the risk of diabetes [10,11,12].

It is known that suicidal ideas and suicide attempts occur more frequently in patients with DM than in the general population [2,13,14,15]. Nonetheless, differences have been reported between patients with type 1 or type 2 diabetes. For instance, a recent study of people who died by suicide in Finland reported that the proportion of patients with type 2 diabetes who completed suicide, was twice the proportion of patients with type 1 diabetes who completed suicide [16]. Other authors indicate that risk factors of suicidal behavior such as depression, anxiety or hopelessness are present in patients with type 2 diabetes [7,17]. However, research about suicidal behavior (SB) risk factors among patients with DM is not very extensive. Thus, the need for further studies to reach conclusive outcomes [16,17,18,19,20].

In Mexico the prevalence of type 2 diabetes massively increased between 2000 and 2015 [21] Suicide attempts have also increased in this period of time [22]. However, to our knowledge, there are no studies that evaluate suicidal behavior in Mexican patients with diabetes. For these reasons, the aims of the present study were: (i) to analyze the prevalence of suicide attempt in patients with type 2 diabetes mellitus, (ii) to investigate the role of demographic and clinical characteristics, anthropometric and biochemical parameters, as well depression, over suicide attempt, and (iii) to determine predictive factors of suicide attempt in patients with type 2 diabetes.

## 2. Materials and Methods

A total of 185 patients with type 2 diabetes were consecutively recruited from the Diabetes Clinic at the “Hospital Regional de Alta Especialidad Dr. Gustavo A. Rovirosa” (in Spanish). All patients were recruited between January and December 2016.

### 2.1. Ethics Statement

This study was approved by the Ethics and Research Committee of the “Hospital Regional de Alta Especialidad Dr. Gustavo A. Rovirosa” (in Spanish) (00228/16). All patients who attended the diabetes clinic were invited to participate. Only patients who received verbal explanations about the study, received the objectives of the study in written and agreed to participate, were included in the study. All patients participated voluntarily and received no monetary compensation. The patients’ participation was independent of the medical attention they usually received at the hospital. Patients who decided to participate signed an informed consent letter that was previously approved by the ethics committee.

### 2.2. Participants

We invited 210 individuals, but only 185 patients complied with the inclusion criteria. The inclusion criteria were: Subjects born in Tabasco State (Mexico). To be 18 year of age and older, have Type 2 diabetes and have a clinical follow-up of at least 1 year in the Diabetes Clinic. Patients with uncontrolled diabetes at moment of the study were also included. Exclusion criteria: patients with incomplete evaluations were eliminated from the study (n = 15).

### 2.3. Characteristics Evaluated

For this study, we designed a questionnaire that included: (a) sociodemographic characteristic of the patients (sex, age, marital status, years of education, use of alcohol and tobacco); (b) anthropometric and biochemical parameters including the time in years since the diagnosis of diabetes was done and (c) glycemic control and use of insulin. Regarding the anthropometric measurements, we assessed body weight and height to determine the body mass index (BMI). We classified the patients as overweight or obese according to cutoff points proposed by the World Health Organization (WHO), following procedures previously reported [23]. The biochemical parameters such as fasting blood glucose, total cholesterol and triglyceride levels were obtained using the clinical chemistry system ERBA XL200-Mannheim (Erba, Mannheim, Germany). Glycated hemoglobin (HbA1c) concentration was determined by colorimetric method. The use of hypoglycemic drugs and insulin were asked as a yes/no question.

Finally, all patients were interviewed to assess their history of suicide attempts, using the Suicide Intent Scale in Spanish version [24]. In Mexican population, the reported Cronbach alpha score is 0.84 [25]. In addition, the Hamilton Depression Rating Scale [26] was used to determine the presence of clinical depression according to a cutoff point of 14, which identifies moderate to severe depression [27]. To measure the emotional distress of living with diabetes, we used the 5-item Problem Areas in Diabetes (PAID-5) in Spanish [28,29]; this questionnaire comprises 5 items, scored on 4-point Likert scale (0–4), with a range from 0 to 20. The points of each item are added; high scores indicated more emotional distress of living with diabetes mellitus.

### 2.4. Statistical Analysis

Demographic and clinical features were analyzed through frequencies and percentages for categorical variables, and through means and standard deviations (S.D.) for continuous variables. Comparative analyses between patients with and without history of suicide attempt were performed using χ^2^ test for categorical variables and independent sample *t*-tests for continuous variables.

The statistical differences from the comparative analyses of clinical variables, were included in a multivariate logistic regression analysis, along with demographic features. To determine the prediction of suicide attempt in patients with diabetes, we used the backward-conditional stepwise selection method. Continuous variables were categorized by a dummy codification using the mean value of the total sample (i.e., age) or according with health parameters (i.e., blood glucose ≤ 125 mg/dL, triglycerides ≤ 200 mg/dL). The Aikake Information Criterion (AIC) was determined to identify the best regression model for the present study, with lower values as indicative of a better model adequacy. Data were analyzed using the statistical software SPSS version 21 for Windows (IBM, Armonk, New York, USA) and significance was established at *p* ≤ 0.05 for all tests.

## 3. Results

A total of 185 patients with type 2 diabetes were included in the study. Women accounted for the majority of the sample (71.4%, n = 132) and the mean age was 54.1 years (S.D. = 13.2, range 18–86). More than half of the patients (61.6%, n = 114) were married at the time of the study. The mean values for biochemical parameters were above the normal values expected for controlled diabetes (see Table 1). Most patients (82.2%, n = 152) were classified as overweight or obese.

### 3.1. History of Suicide Attempt

Previous suicide attempts were reported in 11.4% (n = 21) of patients. Of these, three patients (14.2%) have had one previous suicide attempt, while the remaining patients (n = 18) have had between two and four suicide attempts. Hanging was the most frequent (61.9%, n = 13) suicide attempt method reported, followed by the use of sharp objects (28.6%, n = 6) and firearm weapons (9.5%, n = 2). Patients were then divided into two groups: (1) control group formed by 164 DM patients without suicide attempt history and (2) study group formed by 21 DM patients with suicide attempt history.

### 3.2. Sociodemographic and Clinical Characteristics between Groups

Some significant differences between controls and patients with suicide attempts emerged. Table 1 shows that individuals with a history of suicide attempt were younger (44.6 years, S.D. = 14.0 vs. 55.3 years, 12.6; *p* < 0.001) and less frequently overweight or obese (84.8%, n = 139 vs. 61.9%, n = 13; *p* = 0.01) than those who did not have a history of suicide attempt. A larger number of individuals with suicide attempt history were clinically depressed (33.3%, n = 7 vs. 15.2, n = 25; *p* = 0.03); they also exhibited higher glucose levels in blood (214.2 mg/dL, S.D. = 93.5 vs. 174.4 mg/dL, S.D. = 67.6; *p* = 0.01) and lower triglyceride levels (164.4 vs. 198.8; *p* = 0.01) than those who did not have a history of suicide attempt.

### 3.3. Multivariate Analysis

The logistic regression model included five main explanatory variables for suicide attempt: (1) age, (2) depression, (3) BMI levels, (4) glucose levels and, (5) triglycerides levels, these parameters were categorized with dummy codification as well as age. We decided to include gender as a variable, because it has been observed that women are more prone to suicide attempts than men; then, it may also be an important risk factor in this population. The logistic regression model was adequate according to the Hosmer and Lemeshow value (*p* = 0.53) and correctly classified in 89.2% of the cases. The most important predictors for suicide attempt were a younger age, presence of depression and normal BMI (Table 2). The AIC value obtained in the final regression model showed adequacy compared to the initial AIC value obtained.

## 4. Discussion

Diabetes Mellitus is one of the most psychologically-demanding chronic diseases, where patients show greater loss of motivation, worse future expectations and a decrease in their quality of life. The present study aimed to analyze a Mexican population with T2DM, in order to evaluate the prevalence of suicide attempts, explore possible risks factors of suicide attempt, as well as protective factors.

We observed that the prevalence of suicidal behavior in patients with type 2 diabetes, was higher than the two-fold observed in the Mexican population (11.6% in patients with diabetes versus 5.2% in general population) [30]. Our results were similar to previous reports showing that diabetes increases the risk of death by suicide [9,31].

We found that suicide attempters with T2DM showed higher levels of blood glucose than those without history of suicide. Since glucose is the human brain’s primary source of cellular fuel, psychological processes such as self-control, decision making, and other emotion regulations depend heavily on intracellular availability of glucose in the brain [32,33]. Other reports support our outcomes, for example in 2012 Batty et al., performed a cohort study and observed that the highest suicide rates were apparent in those with type 2 diabetes; furthermore, suicide risk increased when blood glucose levels also increased [1]. A few years later, Chung et al., in a sample of 34,065 subjects reported differences in mental health depending on glucose tolerance statuses and found that depressive mood for two or more continuous weeks, suicidal thoughts, and suicidal attempts, were related to high levels of blood glucose [6]. Nevertheless, when we performed a logistic regression model, glucose levels did not maintain a statistical significance. Therefore, we recommend exploration of this hypothesis in further studies using a larger sample.

We observed that patients in the suicide attempt group, were younger than the control group (44.75 ± 14.01 *p* = 0.001). In this sense, the results of our multiple regression analysis, showed that younger patients had three-fold increase of attempted suicide in comparison to the older population. These data indicate that old age can be a protective factor for suicidal behavior, which is similar to what has been observed in the in the general population, as suicidal behavior prevalence decreases with age [34,35]. The literature suggests that younger individuals have more responsibilities and are under more stress than older individuals [36]. This could be true also for patients with diabetes. Then we could consider age as a comorbidity that influences the development of suicidal behavior from an early age [16]. Then, future studies in suicidal behavior should consider the role of age in patients with type 2 diabetes.

Recently, obesity has been considered as a major risk factor of suicidal behavior; however, the exact link between them remains unclear. Nevertheless, our outcomes revealed an association between normal weight and suicide risk. Other studies have observed that higher BMI is associated with poor health and therefore the individual has maladaptive behaviors that associate with SBs [37,38]. Besides, epidemiological studies with large samples have shown negative associations between BMI and suicidal behavior, where individuals with higher BMI have lower risk of suicide [39,40,41]. These discrepancies could have some explanations. Firstly, gender may be involved as a possible moderator in suicidal behavior; for example, Branco J.C. et al. found a statistical relation between obesity and suicide risk in a group of young adult women, but not in men [42]. Another hypothesis is that glucose homeostasis may cause mental disorders [43,44,45], regardless of the body weight; for example, the group of Bendix M. confirmed that higher insulin and lower glucagon plasma levels, are associated with SB [46]. Hence, we recommend taking into consideration all the clinical and biological factors to understand the nature of SB.

We want to draw attention to the role of mental health. As expected, we observed that depression is a predictor of suicide attempt in patients with type 2 diabetes. Then, we consider that psychologic and psychiatric assessments should be performed in diabetic patients before depressive symptoms appear, which could prevent suicidal behavior [12]. Also, it is necessary to review studies that have evaluated the use of antidepressants in patients with depression and type 2 diabetes [47]. In this sense, a common method of suicide attempt in T2DM patients is use of high doses of insulin and other medications for treating diabetes [7,12]. Therefore, we support a regular screening and prompt treatment of suicidality, or others psychiatric traits in patients with diabetes [48].

On the other hand, we analyzed biochemical variables such as cholesterol levels. Since the 1990’s, cholesterol has been associated with suicide. The hypothesis is that decreased cholesterol in the lipid rafts of synaptic membranes might lead to a reduced of serotonergic neurotransmission involved in suicidal behavior. Nevertheless, studies that have explored this association are methodologically heterogeneous and have produced mixed results [49,50,51]. In our sample, for instance, we did not find any evidence of a significant participation of cholesterol in T2DM patients with suicide attempt. The discrepancies of the outcomes could be due to various causes: these studies did not consider the differences on local dietary habits of the populations that may have an important bearing over cholesterol levels [52,53]. Other reason is that the studies did not consider confounder variables involved in the metabolism of cholesterol [54,55].

We also measured triglyceride levels in our studied population. Patients with type 2 diabetes and suicide attempted showed lower levels of triglycerides than patients without suicide attempt. Our results agree with what has been reported in the literature. A meta-analysis of 65 studies showed that patients with suicidal behavior showed lower levels of triglycerides than not suicidal patients [56]. In contrast, a meta-analysis by Bartoli et al. [57] did not find differences in levels of triglycerides in patients with bipolar disorder and suicide attempt, when compared with non-attempter patients with bipolar disorder. However, in our sample, individuals under 60 years of age showed a significant difference. Then, the role of triglyceride levels as a predictive biomarker of suicidality should be taken with caution, especially because the mechanism that could explain the association between triglycerides and suicide is unknown. More studies are necessary, particularly considering patients with type 2 diabetes. However, to our knowledge, our study is within the first ones in showing that decreased levels of triglycerides are observed in patients with suicide attempt and type 2 diabetes.

To our knowledge, this is the first study that correlates suicide attempt and emotional stress with diabetes mellitus type 2 in a Mexican population. These findings denote that emotional stress related to diabetes is frequent in this population. It is known that in some patients, a poor glycemic control produces emotional stress, while in others, emotional stress obstructs adherence to treatment, leading to poor glycemic control [58]. Our data suggest that emotional stress indirectly causes suicidal behavior, as uncontrolled diabetic patients perceive their illness in a negative way. This causes a vicious cycle unfavorable to their health, in which clinical improvement becomes a challenge for the diabetic patient, and therefore, it causes greater emotional stress [59].

There are several limitations in our study. First, we did not perform an analysis by gender. This was due to the small sample size. Second, the small sample size. In the present study there are numerous factors that appear to be different among individuals with a history of suicide attempt and those without it, which do not reach statistical significance. It is possible that in a larger sample, these differences may be significant and contribute to explaining suicide attempts among individuals with diabetes. Then the need of the more studies considering larger samples are necessary. Third, we performed a cross-sectional study and our patients were evaluated only once; then, cohort studies are necessary to evaluate suicidal behavior in the long term. Fourth, the time and evaluation of suicide attempt. The variables we studied, were not measured around the time when the suicide attempts happened, but after type 2 diabetes was diagnosed. Finally, this study was performed in a population from southern Mexico; therefore, care must be taken when extrapolate the results to the entire Mexican population.

## 5. Conclusions

Our study provided evidence of a high prevalence of suicide attempt in a Mexican population with type 2 diabetes mellitus. On the other hand, we observed that younger age, depression, and normal BMI could be risk factors for suicide attempt in these patients. Then, early psychologic and psychiatric interventions are needed to prevent the presence of depression and suicide attempt. Nevertheless, future studies using larger size samples are necessary to replicate these results.

## Figures and Tables

**Table 1:** Sociodemographic and clinical characteristics of patients with type 2 diabetes with and without suicide attempt.

Variable	Total Sample n = 185		Without Suicide Attempt n = 164		With Suicide Attempt n = 21		Statistics
*Demographic and Clinical Characteristics*							
Age (years)	54.1	13.2	55.3	12.6	44.6	14.0	t = 3.5, *p* < 0.001
Gender—Women	132	71.4%	119	72.6%	13	61.9%	χ^2^ = 1.0, *p* = 0.30
Marital status—Married	114	61.6%	100	61.0%	14	66.7%	χ^2^ = 0.2, *p* = 0.61
Years of education	6.7	4.3	6.7	4.3	7.0	4.3	t = −0.2, *p* = 0.76
Smoking status—Yes	8	4.3%	8	4.9%	-		χ^2^ = 1.0, *p* = 0.30
Alcohol status—Yes	36	19.5%	35	21.3%	1	4.8%	χ^2^ = 3.2, *p* = 0.07
Hypoglycemic drug use—Yes	171	92.2%	153	93.3%	18	85.7%	χ^2^ = 1.6, *p* = 0.44
Insulin use—Yes	140	75.6%	123	75.0%	17	81.0%	χ^2^ = 0.5, *p* = 0.77
*Anthropometric Measurements*							
Weight (kg)	74.8	19.4	75.2	19.6	72.2	18.1	t = 0.6, *p* = 0.51
Height (cm)	156.2	9.4	155.9	9.5	158.9	8.3	t = −1.3, *p* = 0.17
Body mass index (BMI)	30.8	9.0	31.1	9.2	28.7	7.7	t = 1.1, *p* = 0.25
BMI—Normal	33	17.8%	25	13.2%	8	38.1%	χ^2^ = 6.6, *p* = 0.01
*Biochemical Measurements*							
Glucose level in blood (mg/dL)	179.0	71.8	174.4	67.6	214.2	93.5	t = −2.4, *p* = 0.01
HbA1c (%)	8.8	2.3	8.7	2.2	9.7	2.6	t = −1.8, *p* = 0.06
Total cholesterol (mg/dL)	193.5	45.9	195.0	47.6	181.9	27.7	t = 1.2, *p* = 0.07
Triglycerides (mg/dL)	194.9	102.5	198.8	107.0	164.4	46.8	t = 1.4, *p* = 0.01
*Depression and Problem Areas in Diabetes*							
Hamilton Scale	7.7	7.5	7.0	7.1	13.3	8.4	t = −3.7, *p* < 0.001
Depression—Yes	32	17.3%	25	15.2%	7	33.3%	χ^2^ = 4.2, *p* = 0.03
PAID-5	5.1	4.9	4.8	4.9	7.1	4.7	t = −1, *p* = 0.052

Data are in mean (S.D.) or n (%).

**Table 2:** Predictors of suicide attempt in patients with type 2 diabetes mellitus.

**Initial Regression Model AIC Value: 68.60**					
**Variable**	**β**	**SE β**	**Odds Ratio**	**95% CI**	***p*-Value**
Younger age	1.18	0.53	3.27	1.14–9.41	0.02
Gender-Female	0.33	0.36	1.39	0.47–4.05	0.54
Depression-Present	1.21	0.57	3.37	1.09–10.36	0.03
Normal BMI Level	1.00	0.55	2.73	0.92–8.10	0.06
Lower Triglyceride Levels	0.51	0.56	1.68	0.55–5.07	0.35
Higher Glucose Levels	0.80	1.04	2.24	0.47–10.61	0.30
**Final Regression Model AIC Value: 30.29**					
	**β**	**SE β**	**Odds Ratio**	**95% CI**	***p*-Value**
Younger age	1.29	0.52	3.63	1.29–10.19	0.01
Depression	1.20	0.54	3.33	1.13–9.76	0.02
Normal BMI Level	1.14	0.52	3.14	1.11–8.83	0.03

Value used to perform dummy codification on continuous variables: Age (mean value: 54 years old), Depression (cutoff score of 14 in the Hamilton Depression Rating Scale), BMI Levels (Normal BMI < 25), Triglycerides (≤200 mg/dL) Blood Glucose (≥125 mg/dL).
